# System Design Considerations for Magneto‐Electrocatalysis of the Oxygen Evolution Reaction

**DOI:** 10.1002/smll.202500001

**Published:** 2025-03-16

**Authors:** Dorottya Szalay, Amy Radford, Yiyang Li, Shik Chi Edman Tsang

**Affiliations:** ^1^ Wolfson Catalysis Centre, Department of Chemistry University of Oxford Oxford OX1 3QR UK

**Keywords:** lorentz force, magnetohydrodynamics, magnetoresistance, spin‐pinning, water‐splitting

## Abstract

The integration of an external magnetic field into electrocatalysis, termed magneto‐electrocatalysis, can target efficiency challenges in the oxygen evolution reaction (OER). Reaction rates can be enhanced through improved mass transport of reactants and products, manipulation of spin states, and lowered resistance. The OER is a kinetic bottleneck in electrocatalytic water splitting for sustainable hydrogen fuel. Previous studies lack comprehensive analyses and consistent reporting of magnetic field effects, resulting in varied interpretations. To establish optimized and reliable systems at larger scales, significant research advancements are required. This perspective explores the complex impact of magnetic fields on OER, emphasizing the interplay between various mechanisms such as spin‐polarization of oxygen intermediates, Lorentz force‐induced magnetohydrodynamics, and magnetoresistance. Here, how experimental design – such as electrode magnetism, shape, positioning, and reactor setup – can significantly influence these mechanisms is highlighted. Through a comprehensive review of current studies, major knowledge gaps and propose methodologies are identified to improve experimental reproducibility and comparability. This article aims to guide researchers toward the development of more efficient, scalable systems that leverage magnetic fields to enhance water splitting to push forward commercial green hydrogen production.

## Graphical abstract

Electrocatalytic water‐splitting can be controlled using a magnetic field. The commercial prospects are exciting, but the progress is stuck in the fundamental stage of research. Progress is stagnated by publications with conflicting results, incomplete analysis, and inconsistent experimental setups. In this Perspective, we discuss the need for reform in experimental design and analytical procedures, to allow this technique to move forward.

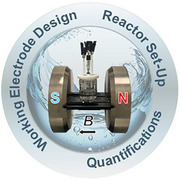

## 1 Introduction

Magneto‐electrocatalytic research progress is stagnated by publications reporting conflicting results with incomplete analysis. Comparison between studies is made even more difficult by inconsistent experimental setups. Only with improvements in the research methodology can advancements toward optimized magnetic enhancements be made, in pursuit of the commercial scale‐up of the magneto‐electrocatalytic water‐splitting process.

To improve the efficiency of electrocatalytic water‐splitting, utilizing static magnetic fields is among several promising strategies that make use of external stimuli, such as light, heat, or pressure.^[^ ^1^, ^2^, ^3^, ^4^ ^]^ Designing systems that can reach higher performances in the water‐splitting reaction is vital in the pursuit of affordable green H_2_. With a high energy density of 143 kJ kg^−1^ – approximately three times that of liquid hydrocarbon – and no greenhouse gas emissions on combustion, H_2_ is an ideal green fuel.^[^ ^5^ ^]^

Electrochemical water splitting consists of two half‐reactions: the hydrogen evolution reaction (HER) at the cathode and the oxygen evolution reaction (OER) at the anode, both of which are pH‐dependent reactions.^[^ ^6^ ^]^ OER is generally considered to be the bottleneck of overall water‐splitting. While HER is only a 2‐electron process, OER involves the transfer of 4‐electrons in both acidic (Equation 1) and alkaline media (Equation 2), resulting in sluggish kinetics and high overpotentials.^[^ ^7^ ^]^

(1) $$2H_{2}O\to 4H^{+}+4e^{-}+O_{2}$$

(2) $$4OH^{-}\to 2H_{2}O+4e^{-}+O_{2}$$

Being identified as a key efficiency‐limiter, OER has become a huge topic in the scientific research community. Today, the benchmark catalysts for electrochemical OER are IrO_2_ and RuO_2_, due to their ideal adsorption energies of key intermediates giving low overpotentials.^[^ ^8^ ^]^ Although these precious metals demonstrate excellent performance, their large‐scale use is limited by high energy costs and their scarcity, making them economically and environmentally unsustainable.^[^ ^9^, ^10^ ^]^ Transition metal catalysts are seen as a cheaper alternative but cannot yet reach the efficiencies set by their precious metal counterparts. To improve the catalytic activities of materials, strategies include: morphology engineering (where the catalyst's microstructure is altered)^,^ use of various support materials, and chemical doping.^[^ ^11^, ^12^, ^13^, ^14^, ^15^, ^16^ ^]^

**Figure 1:**
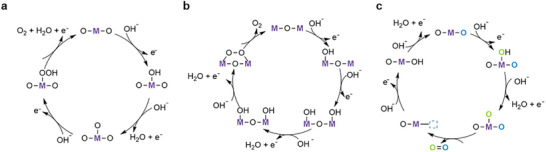
OER mechanisms in alkaline media for metal oxide catalysts. a) conventional AEM mechanism steps b) OPM mechanism steps and c) LOM mechanism steps. M represents the active metal sites.

The process of electrocatalytic reactions can be summarized into three major steps: 1) mass transport of reagents to the electrode surface, 2) reaction at the electrode, and 3) mass transport of products away from the electrode.^[^ ^17^, ^18^, ^19^ ^]^ It is generally agreed that the second step (reaction at electrode) of alkaline OER can proceed through one of three pathways: the conventional adsorbate evolution mechanism (AEM, **Figure** **1**a), the oxide path mechanism (OPM, Figure 1b), or the lattice oxygen mechanism (LOM, Figure 1c).^[^ ^20^ ^]^ Each involves the transfer of 4‐electrons but differs in the intermediates and the structural requirements of the active sites.

Magnetic fields have emerged as a particularly promising strategy for enhancing OER because they can target the three major reaction steps of electrocatalysis.^[^ ^21^ ^]^ Electrode spin polarization and Lorentz force create effects such as magnetohydrodynamics, Hall effect, magnetoresistance, and triplet O_2_ (^3^O_2_) promotion that have been pinpointed as the key mechanisms influencing magneto‐electric water splitting mechanisms. Kelvin force and Maxwell stress can also be present in magneto‐electrocatalysis but are less significant in water‐splitting.

The type of magnetic field effect in a given system is dictated by the magneto‐electric mechanisms it favors. Thus, the literature reports a mix of positive and negative magnetic effects of different magnitudes, depending on the characteristics of the electrocatalytic system. The mechanisms depend on factors such as the magnitude and direction of the external magnetic field, the shape and composition of the electrodes, and the dimensions of the reactor.^[^ ^22^, ^23^ ^]^ Unfortunately, the research in this field lacks cohesion, making it difficult to draw detailed conclusions on how experimental parameters can be controlled to favor specific mechanisms.

In this *Perspective*, we highlight the necessity of deliberate and sensible experimental design. By increasing the number of reliable, repeatable, and properly analyzed reports, we can begin to truly unravel the core chemical mechanisms and interactions governing magneto‐electrochemical OER. After breaking down the major magneto‐electric mechanisms, we explore how experimental design impacts the type of mechanisms presented and the size of the magnetic effect produced. To address the well‐studied yet controversial nature of this field, we identify research gaps in the existing literature and propose experimental strategies that could solidify conclusions from prior studies, advancing the field significantly.

## 2 Magneto‐Electrocatalytic Mechanisms

### 2.1 Lorentz Force and Magnetohydrodynamics

**Figure 2:**
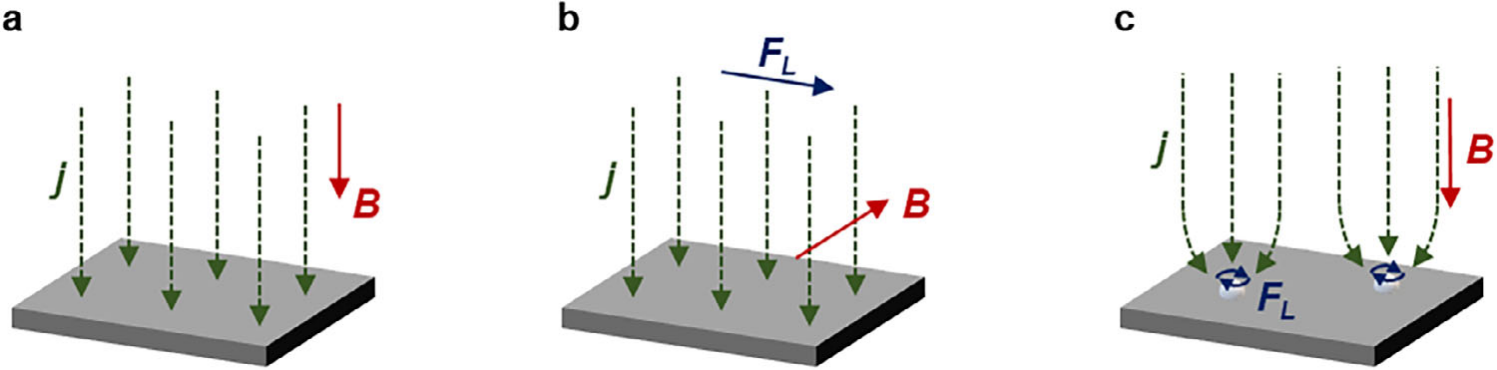
Lorentz force effect when a) *j* is parallel to *B* and *F_L_* is not present, b) *j* is perpendicular to *B* and *F_L_* is at a maximum, and c) *j* is parallel to *B* but uneven electrode surface creates a bend in *j* causing small *F_L_*.

Lorentz force and its effects are among the most commonly discussed contributors to magneto‐electrochemistry. Lorentz force acts on charged particles under an external magnetic field and is described by Equation (3):

(3) $$F_{L}=j\times B$$

where *j* is current density and *B* is magnetic flux. The Lorentz force is zero when *j* and *B* are parallel (**Figure** **2**a) and maximized when *j* and *B* are perpendicular (Figure 2b). The direction of Lorentz force is perpendicular to the co‐plane formed by *B* and *j*. It is also important to discuss Lorentz force effects in cases where the direction of current flow between two electrodes is parallel to *B*. This is due to the bending of the electric field on even electrode surfaces, electrode edges, and adsorbed bubbles, resulting in a small but significant Lorentz force (Figure 2c).^[^ ^24^ ^]^

In an electrocatalytic system, the Lorentz force can act on charged particles to create macro‐level convections in the electrolyte. These convections are referred to as magnetohydrodynamic (MHD) effects.^[^ ^25^ ^]^ MHD is strongly dependent on the size and shape of electrodes, the distances between them, and reactor dimensions. This convection can the mass transport reaction steps by impeding or promoting bubble release and thinning the diffusion layer at the electrode surface.^[^ ^22^, ^24^, ^26^, ^27^, ^28^ ^]^

### 2.2 Magnetoresistance

The magnetoresistance (MR) effect is often overlooked in magneto‐electrocatalysis. It describes how the electrical resistance of metals or semiconductors changes in response to an applied magnetic field. Defined by Equation (4):

(4) $$MR=\frac{R_{B}-R_{0}}{R_{0}}$$

where *R_B_* is electrical resistance under the effective magnetic field (*B*) and *R_0_* is resistance without an external magnetic field. If a material's electrical resistance increases under an applied magnetic field (*R_B_* > *R_0_*), MR will have a positive value. When this resistance decreases under H (*R_B_* < *R_0_*), MR is negative.^[^ ^29^ ^]^ The effect can arise from spin‐polarization; where negative MR (nMR) can result from facilitated electron transport through spin‐channels in highly magnetized materials, and positive MR (pMR) can consequence from the Zeeman effect that increases spin‐disorder scattering through spin‐mixing. Lorentz force also creates positive MR by bending the flow of charge carriers moving through a material – the Hall effect.^[^ ^30^, ^31^, ^32^ ^]^ In electrocatalysis, MR in the electrode will target the electron transfer steps of the reaction by affecting charge‐carrier availability, and may also target the diffusion of charged species in the electrolyte by affecting the polarization of the electrode.

### 2.3 Spin‐Pinning

**Figure 3:**
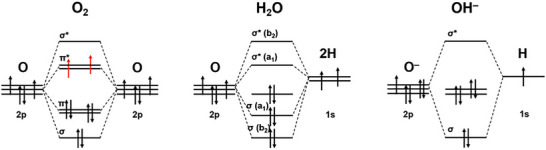
MO diagrams of O_2_, H_2_O, and OH^−^. Reproduced with permission.^[^ ^33^ ^]^ Copyright 2020, Wiley.

OER in both alkaline and acidic media involves spin‐dependent electron transfers.^[^ ^33^ ^]^ As illustrated in the molecular orbital (MO) diagrams (**Figure** **3**), the oxygen sources – OH^−^ in alkaline media and H₂O in acidic media – are in singlet states, meaning their valence electrons are fully paired (diamagnetic). In contrast, the O₂ product exists predominantly in the thermodynamically‐favored triplet ground state, characterized by two unpaired electrons (↑O = O↑).^[^ ^34^ ^]^ The singlet excited state of O₂ (↑O = O↓) lies ≈1 eV higher in energy than the triplet ground state.^[^ ^35^ ^]^

**Figure 4:**
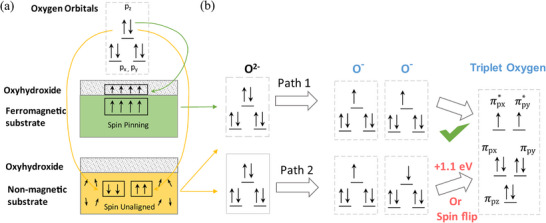
“Spin‐pinning” effect for triplet oxygen formation on an oxyhydroxide surface. a) The spin‐electron transfer from singlet oxygen (OH^−^, H_2_O) to Co_3−x_Fe_x_O_4_/ Co(Fe)O_x_H_y_ with and without spin pinning effect. b) The triplet oxygen production by two oxygen radicals in parallel/opposite spin alignments. Reproduced with permission.^[^ ^34^ ^]^ Copyright 2021, Creative Commons Attribution 4.0 International License.

Several studies have proposed that the change in the spin state of oxygen species (going from singlet starting materials to triplet products) can significantly contribute to the sluggish kinetics and high overpotentials associated with OER.^[^ ^36^, ^37^ ^]^ Under an external magnetic field, alignment of unpaired spins on the active site of the catalyst has been shown to “pin” the spins of oxygen species during O─O bond formation, resulting in the formation of the more energetically favorable triplet molecular oxygen (**Figure** **4**).^[^ ^34^, ^38^, ^39^, ^40^ ^]^

### 2.4 Kelvin Force

Kelvin force acts on paramagnetic species dissolved in the electrolyte in non‐uniform magnetic fields. This force draws the paramagnetic species into areas with a higher magnitude of the magnetic field. Therefore, through the movement of species in the electrolyte, Kelvin force can create convection. Although in most alkaline magneto‐electrocatalytic OER systems, Lorentz force is the main cause of magnetohydrodynamics. While Lorentz force dominates charged species in uniform fields, Kelvin force acts on paramagnetic species in a non‐uniform magnetic field.^[^ ^41^ ^]^ The low concentration of paramagnetic electrolyte species present in electrocatalytic water‐splitting likely makes Kelvin force a relatively weak contributor to MHD. It is possible, however, for Kelvin force to act on paramagnetic ^3^O_2_, attracting it to a magnetic electrode and hindering product diffusion – although this has not yet been properly studied for OER. The force is predominantly studied on electrodeposition using electrolytes with high concentrations of paramagnetic species such as Ni^2+^ and Cu^2+^.^[^ ^42^, ^43^ ^]^

### 2.5 Maxwell Stress

Maxwell stress describes the surface stress on a species with a dipole moment in the electrolyte under a uniform magnetic field. Maxwell stress can lead to a distortion in the shape of these paramagnetic species.^[^ ^44^, ^45^ ^]^ If the electric double layer – the structure of ions and solvent molecules that form on electrode/electrolyte interface – contains paramagnetic species, it can be distorted under a magnetic field by Maxwell stress.^[^ ^45^ ^]^ This can lead to a change in electrochemical double‐layer capacitance (C_dl_), which is directly proportional to the electrochemical surface area (ECSA). An increase in C_dl_ would lead to an increased ECSA that should be beneficial for catalytic activity. As mentioned above, the low concentration of paramagnetic electrolyte species in water‐splitting is likely to make the effects of Maxwell stress very small. It is also important to note that Maxwell stress effects on solid electrodes are negligible as these have a fixed shape, but species in the electrolyte are more elastic.^[^ ^44^ ^]^

## 3 Experimental Design and the Magnetic Effects

In this section, we discuss important aspects of designing magneto‐electrocatalytic reactions on a laboratory scale. Discrepancies between studies make it hard to compare the effects of various catalytic systems and therefore, makes it hard to understand why certain system parameters lead to different effects. By improving experimental designs and with more systematic analysis, we can make our research more cohesive that will allow the field to progress faster.

### 3.1 Working Electrode Composition

Here, we discuss working electrode (WE) design for ME experiments with a focus on the materials’ magnetic properties and magnetoresistance.

The magnetic properties of catalysts used in OER can vary between diamagnetic, paramagnetic, ferromagnetic, antiferromagnetic, ferrimagnetic, and superparamagnetic. The property is determined by the electron spins within the material that determines how it responds to an external magnetic field.

Diamagnets have no unpaired spins and therefore present no net magnetic dipole moment. The magnetic susceptibilities – how much the material becomes magnetized in response to an external magnetic field – of diamagnets are negative and relatively small compared to other types of magnetism.^[^ ^58^, ^59^ ^]^

Paramagnetic materials have unpaired electrons; however, these are randomly oriented and therefore the material does not show magnetism without an external field. When an external field is applied, these randomly oriented spins align parallel with the field and the material shows bulk magnetization.^[^ ^59^ ^]^

Ferromagnetic materials form magnetic domains within the structure. The unpaired spins within these domains are all aligned with each other. Throughout the material, the domains can cancel each other, meaning the materials are not necessarily magnetized in the absence of an external field. On application of an external magnetic field, the spins of individual domains align across the material to create significant magnetization.^[^ ^60^ ^]^

Three less common types of magnetism can also be present for certain catalysts: antiferromagnetism, ferrimagnetism, and superparamagnetism. Similar to ferromagnets, both antiferromagnets and ferrimagnets possess magnetic domains. Within an antiferromagnetic domain, neighboring unpaired spins have opposing directions and therefore the domains present no net magnetization. In ferrimagnetic domains, both parallel and antiparallel spins are present but there is an excess of parallel spins and therefore the domain does have net magnetization. If the size of a ferro‐ or ferrimagnetic particle is decreased to the point where the whole particle is a single domain, superparamagnetism is present with unique, temperature‐dependent properties.^[^ ^59^ ^]^

**Figure 5:**
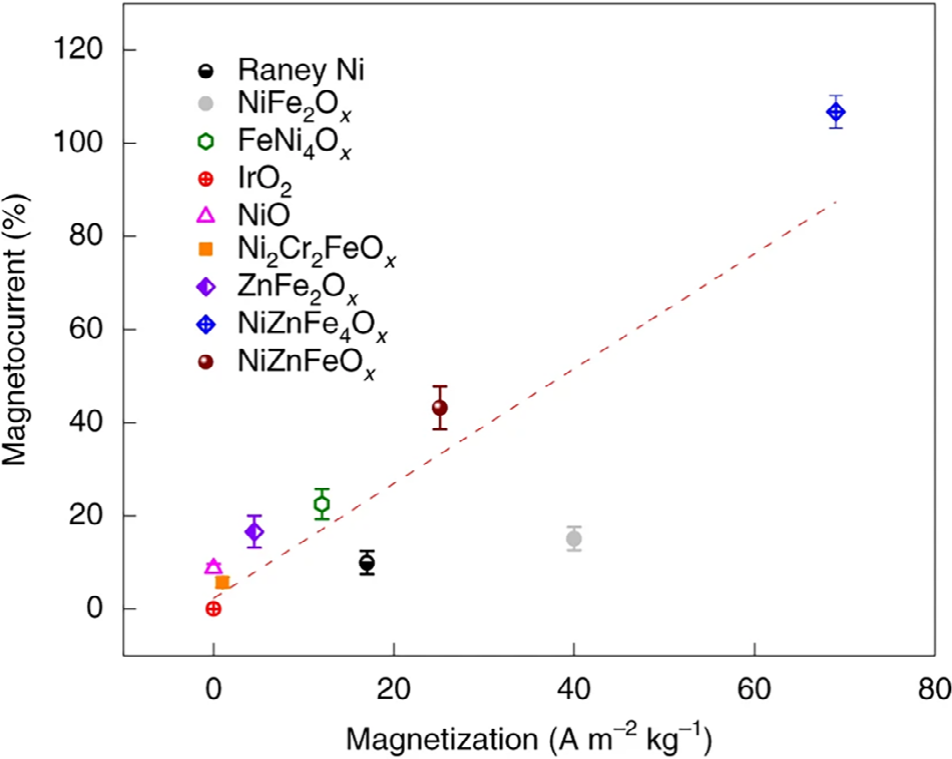
Relationship between catalysts’ bulk magnetization and observed magneto‐current effects for a wide range of catalysts at 1.67 v versus RHE under 450 mT field. Catalysts were deposited on Ni foil. Reproduced with permission.^[^ ^38^ ^]^ Copyright 2019, Springer Nature.

In a pioneering study by Garcés‐Pineda et al. in 2019 – work that has been widely referenced in subsequent publications in the field – a wide range of mostly nickel‐based catalysts was investigated. The authors linked increasing bulk magnetization to an increasing magneto‐current, which was defined as a percentage change between a magnetic field on and a magnetic field off current densities (**Figure** **5**). The benchmark catalyst, weakly paramagnetic IrO_2_, showed negligible response to the external magnetic field. A linear relationship was observed, with NiFe_2_O_x_ being the only exception. For NiFe_2_O_x_, the lower magneto‐current was hypothesized to result from a difference in bulk and surface magnetization of the material.^[^ ^38^ ^]^

**Figure 6:**
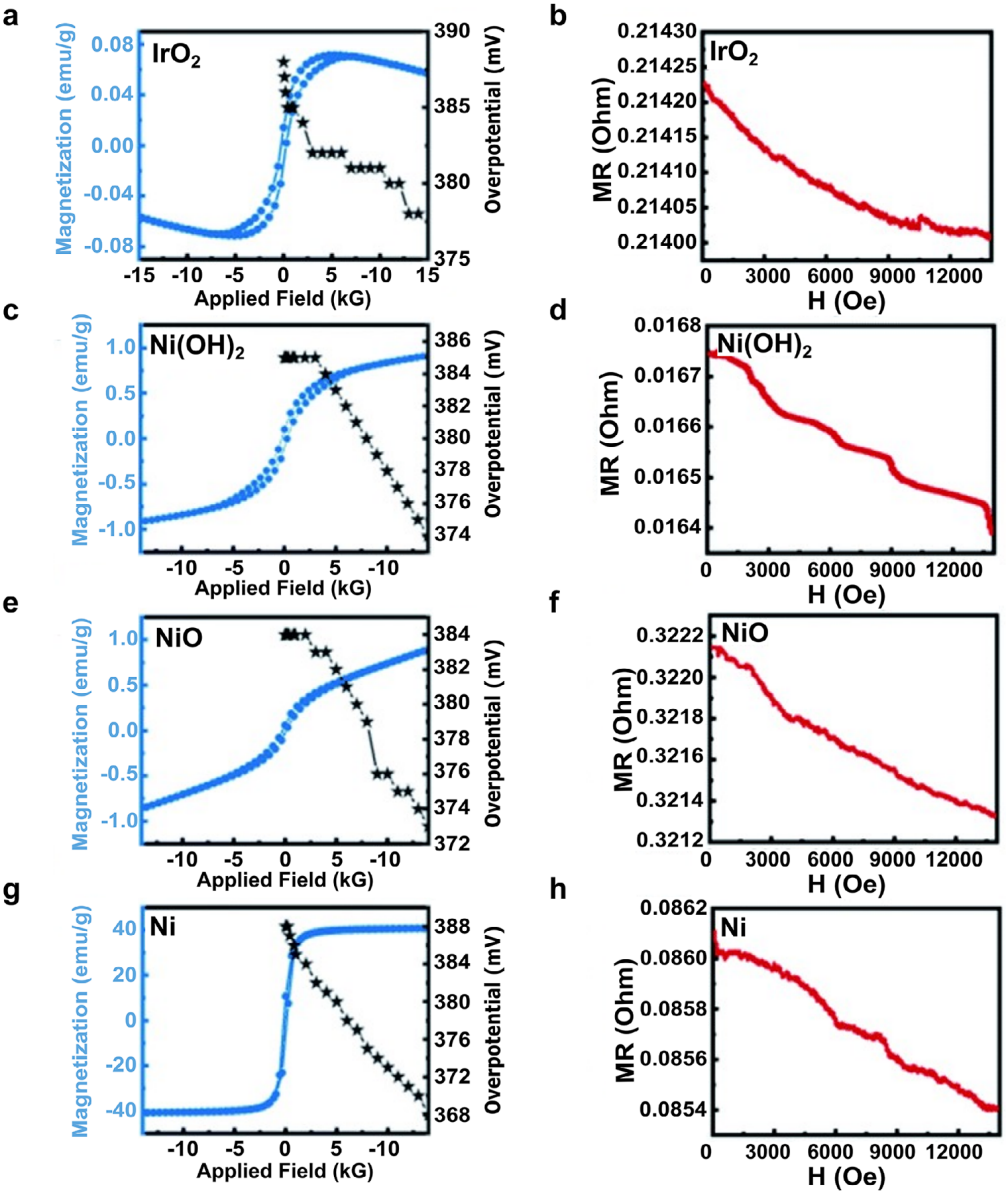
Magnetic hysteresis loops and OER overpotentials (at 10 mA cm^−2^) and magnetoresistance of IrO_2_ a,b), Ni(OH)_2_ c,d), NiO e,f), and Ni g,h) under magnetic field from 0 to 14 000 G. Reproduced with permission.^[^ ^29^ ^]^ Copyright 2022, Royal Society of Chemistry.

Zhang et al. reported lowered OER overpotential (at 10 mA cm^−2^) for paramagnetic IrO_2_ and various antiferromagnetic and ferromagnetic nickel‐based materials using fields up to 1.4 T.^[^ ^29^ ^]^ nMR was identified in the magnetic materials and attributed this effect to the improved OER performance. The authors also considered MHD that was visually observed but dismissed it as a dominating effect, as the overpotential did not drop back to initial levels after the magnetic field was removed. While these effects will act simultaneously, the authors have attributed the performance improvements to nMR, based on the consistent relationship between the change in MR and electrochemical activities (**Figure** **6**). Nickel is often used as a substrate for the active catalyst in working electrodes (**Table** **1**), and therefore the nMR of the substrate material could enhance the performance of the catalyst by reducing the resistance of the system. We want to highlight the importance of considering and investigating magnetic properties (magnetism, magnetoresistance) of not only the catalyst but the substrate as well.

**Table 1:** Summary of recent literature, the magneto‐electrochemical set‐ups used, and main findings for OER. Some abbreviations used in the table are detailed below it.

Catalyst	Substrate	CE	RE	Electrolyte	Magnet	Field strength	Set‐up details	Main findings	Refs.
Range of metal oxides^a)^	Ni foil, Ni foam, or FTO	Pt mesh	Ag/AgCl	1 m KOH	One Nd magnet ring	0–450 mT	Only applied to WE	Current density increased by over 100% for highly magnetic catalysts	[38]
CoFe_2_O_4_, Co_3_O_4_, IrO_2_	Glassy carbon	Pt foil	Hg/HgO	1 m KOH	Not described	0–1 T	Not described	Tafel slope decreased from ≈120 to ≈90 mVdec^−1^ No change for non‐ferromagnetic catalysts	[46]
Metallic carbon nanodots	Glassy carbon (RDE)	Pt/C	Saturated calomel electrode	1 m KOH	Stack of little magnets	0–350 mT	Magnets placed inside the electrolyte	Overpotential decreased by 15 mV @ 10 mA cm^−2^	[47]
Ni(OH)_2_, NiO, IrO_2_ and Ni foam	Carbon cloth	Graphite rod	Hg/HgO	1 m KOH	Vibrating magnetometer	0–1.4T	Entire reactor inside a magnet	Ni: overpotential decreased by 20 mV @ 10 mA cm^−2^	[29]
La_1‐x_Sr_x_MnO_3_	Glassy carbon	Pt plate	Ag/AgCl	1 m KOH	Electromagnet	0–1 T	Entire reactor inside a magnet	Significant nMR contribution Overpotential decreased by 120 mV @ 10 mA cm^−2^	[48]
NiFe‐LDH/Co_3_O_4_	Ni foam	Graphite rod	Hg/HgO	1 m KOH	Vibrating magnetometer	0–1 T	Entire reactor inside magnet	Overpotential decreased by 25 mV @ 10 mA cm^−2^	[49]
NiFe thin films	Si	Pt foil	Hg/HgO	1 m KOH	Electromagnet	200 mT	H‐cell, the field only applied to WE	Multi‐domain structure turns into a single‐domain Overpotential decrease by ≈16 mV @ 10 mA cm^−2^	[50]
Ni, Pt, graphite	–	Ni, Pt, graphite	–	5–50% KOH	RbFe iron magnets	0–4.5 T	Field perpendicular to current density	Ni: current density increases by 14.6% @ 4 V Pt: current density increases by 10% @ 4 V	[22]
Ni wire	–	Ni wire	RHE	2 m KOH	Nd magnet bars	0–1 T	Field perpendicular to current density	Downward Lorentz force resulted in lowest overpotentials	[28]
Ni foam	–	Pt sheet	Hg/HgO	0.1 m and 1 m KOH	Electromagnet	0–200 mT	Entire reactor inside a magnet	Overpotential decreased by ≈200 mV	[51]
Pt, Au	–	Pt mesh	SHRE	1 m KOH	Electromagnet	0–550 mT	H‐cell, the field only applied to WE	Marginal enhancement for non‐magnetic catalysts	[52]
Co_3_O_4_	Ni foam	Pt foil	Ag/AgCl	1 m KOH	Electromagnet	0–125 mT	Entire reactor inside a magnet	Overpotential decreased by 56 mV@ 20 mA cm^−2^	[53]
CoO_x_	FTO	Pt wire/mesh	Ag/AgCl	0.1 m KOH	One NdFeB magnet	0–371 mT	Magnet on a linear actuator	Current density increased by 4.7% @ 1.5 V vs NHE	[37]
Co_3‐x_Fe_x_O_4_ (sulfurized)	Glassy carbon	Pt plate	Hg/HgO	1 m KOH	Not described	0–500 mT	Not described	Spin‐pinning increased activity by ≈1 order of magnitude	[34]
CoFe_2_O_4_, Co_3_O_4_, Fe_3_O_4_, Fe_3_O_4_@NiOOH	Carbon paper	Pt	Hg/HgO	1 m KOH	Electromagnet	Field > FM catalysts’ coercivity	H‐cell, the field only applied to WE	Reducing FM domain size can lead to the same result as applying external field	[54]
IrO_2_	L‐shaped glassy carbon	Graphite rod	Saturated calomel electrode	0.1 m HClO_4_, 0.1 m KOH, 0.1 m KHCO_3_	Electromagnet	0–530 mT	3 compartment cell, the field is only applied to WE	External field does not influence OER	[55]
NiFe LDH	RDE	Graphite rod	Ag/AgCl	1 m KOH	NdFeB magnets	0–300 mT	Magnets placed inside the electrolyte	Overpotential decreased by 42 mV @ 10 mA cm^−2^ under	[56]
CoO_x_	FTO	Pt sheet	Ag/AgCl	0.1 m KOH	Permanent magnet	0–400 mT	Field applied to the back of WE	Overpotential decreased by 65 mV @ 10 mA cm^−2^ O_2_ yield increased 1.4x at pH 10	[36]
M_1_/MoS_2_, IrO_2_	Cu foam	Graphite rod	Hg/Hg_2_Cl_2_	1 m KOH	10 or 100 Nd magnets stacked	0–500 mT	Field applied to the back of WE	Current density between 1.5–1.8 V increased by ≈5.2–11.7 times	[39]
Ni_4_FeO_x_	FTO	Pt mesh	Hg/HgO	1 m KOH	Two permanent magnet bars	0–30 mT	Reactor is placed in between two magnets	Onset potential decreased by ≈20 mV Current density increased by ≈20% above 1.6 V vs RHE	[57]

^a)^ These metal oxides are: NiFe_2_O_x_, FeNi_4_O_x_, Ni_2_Cr_2_FeO_x_, IrO_2_, NiO, ZnFe_2_O_x_, NiZnFe_4_O_x_, NiZnFeO_x_.

CE, counter electrode; RE, reference electrode; RDE, rotating disk electrode; FTO, fluorine‐doped tin oxide; SHRE, standard hydrogen reference electrode.

Ren et al. studied three catalysts: CoFe_2_O_4_ (ferromagnet), Co_3_O_4_ (antiferromagnet) and IrO_2_ (weakly paramagnetic). They found that OER under an external magnetic field was only enhanced for ferromagnetic CoFe_2_O_4_. This was proposed to relate to this catalyst's ability to polarize the spins of adsorbed oxygen intermediates leading to faster kinetics of triplet O_2_ formation (as discussed above in Section 2.3). The team used density functional theory (DFT) calculations to show a change in CoFe_2_O_4_ spin density leading to enhanced kinetics of spin‐charge transfer.^[^ ^46^ ^]^ It's unclear, however, if the observations for this catalyst series relate to the magnetism or the specific atomic and electronic structures. We should also note that these results contradict the findings from Zhang et al. who show improvements in OER for an IrO_2_ catalyst under similar magnetic field conditions.^[^ ^29^ ^]^

**Figure 7:**
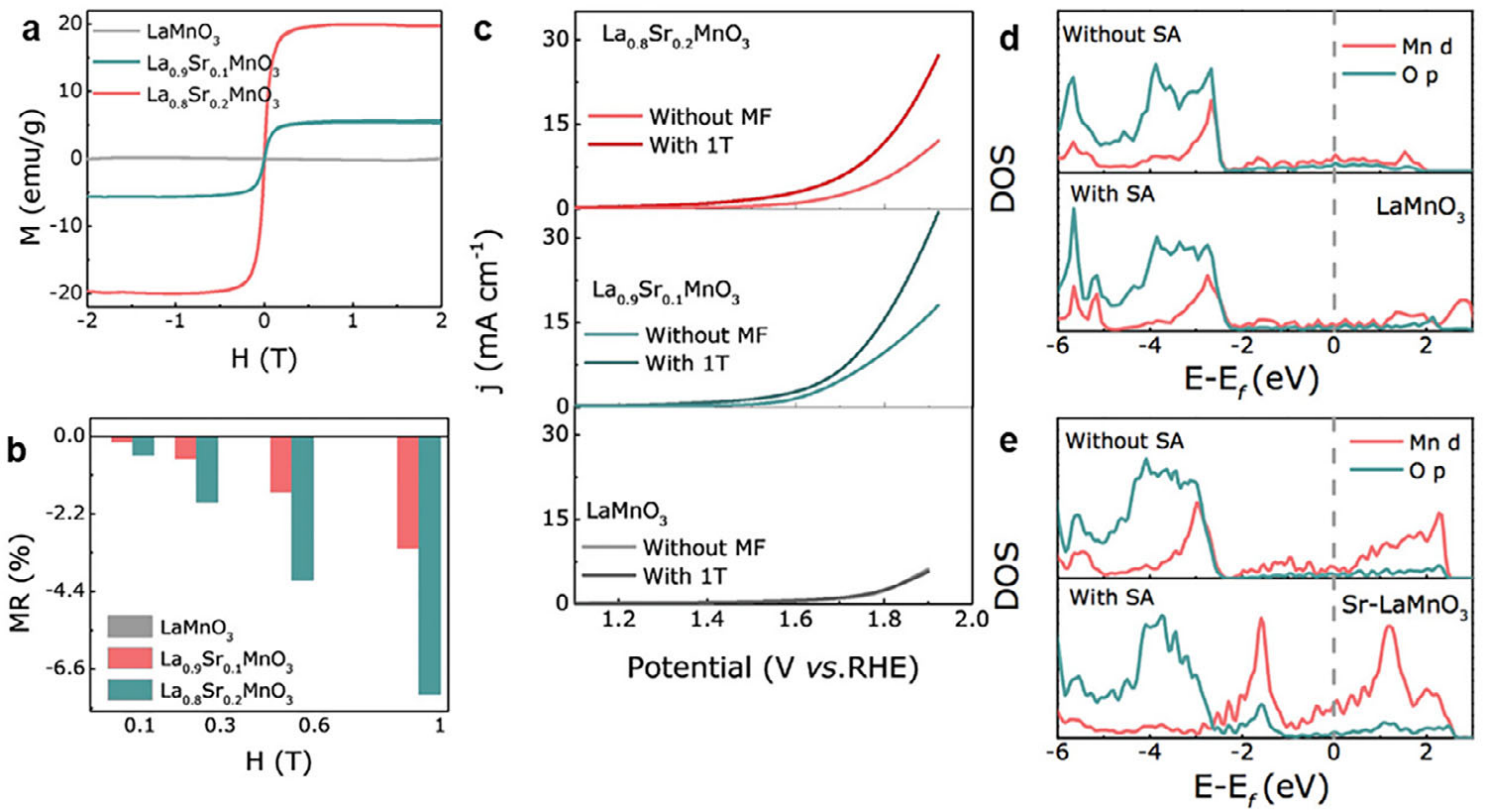
a) Magnetization as a function of applied field between −2 and 2 T for La_1—x_Sr_x_MnO_3_ samples. b) Percentage magnetoresistance values for the same 3 samples at increasing fields. c) LSV curves with and without 1 T field. Reproduced with permission.^[^ ^48^ ^]^ Copyright 2023, Wiley.

Qian et al. successfully doped LaMnO_3_ – an insulator with antiferromagnetic properties – with Sr to create ferromagnetic half‐metal catalysts (**Figure** **7**a). Under a magnetic field, the MR of the two Sr‐modified samples decreased significantly, but the undoped sample showed negligible change in MR (Figure 7b). While undoped LaMnO_3_ showed no change in OER performance under a magnetic field, the two ferromagnetic catalysts showed improved performance; attributed to increased spin polarization and significant nMR. Under a 1.0 T field, a significant nMR of −7.32% for ferromagnetic La_0.8_Sr_0.2_MnO_3_ was proposed, to decrease the charge transfer resistance that caused a 120 mV drop for OER (Figure 7c). Spin polarization was also studied by DFT calculations. The team found a negligible change in the projected density of states (PDOS) for the antiferromagnetic pristine material. However, there was a clear increase in the PDOS near the Fermi level in the ferromagnetic state, contributing to the enhancement of spin‐selected charge transport and improved OER kinetics (Figure 7d,e).^[^ ^48^ ^]^

Zhang et al. made an interesting proposal that the nMR of the nickel foam core in a NiFe‐LDH/Co_3_O_4_/NF electrode was proposed to contribute to a 25 mV overpotential drop under a 1.0 T field.^[^ ^49^ ^]^ This idea could be controversial as catalysis is generally assumed to be a surface‐sensitive reaction, however, a comparison is made with a carbon cloth core where negligible magnetic effect is observed.

**Figure 8:**
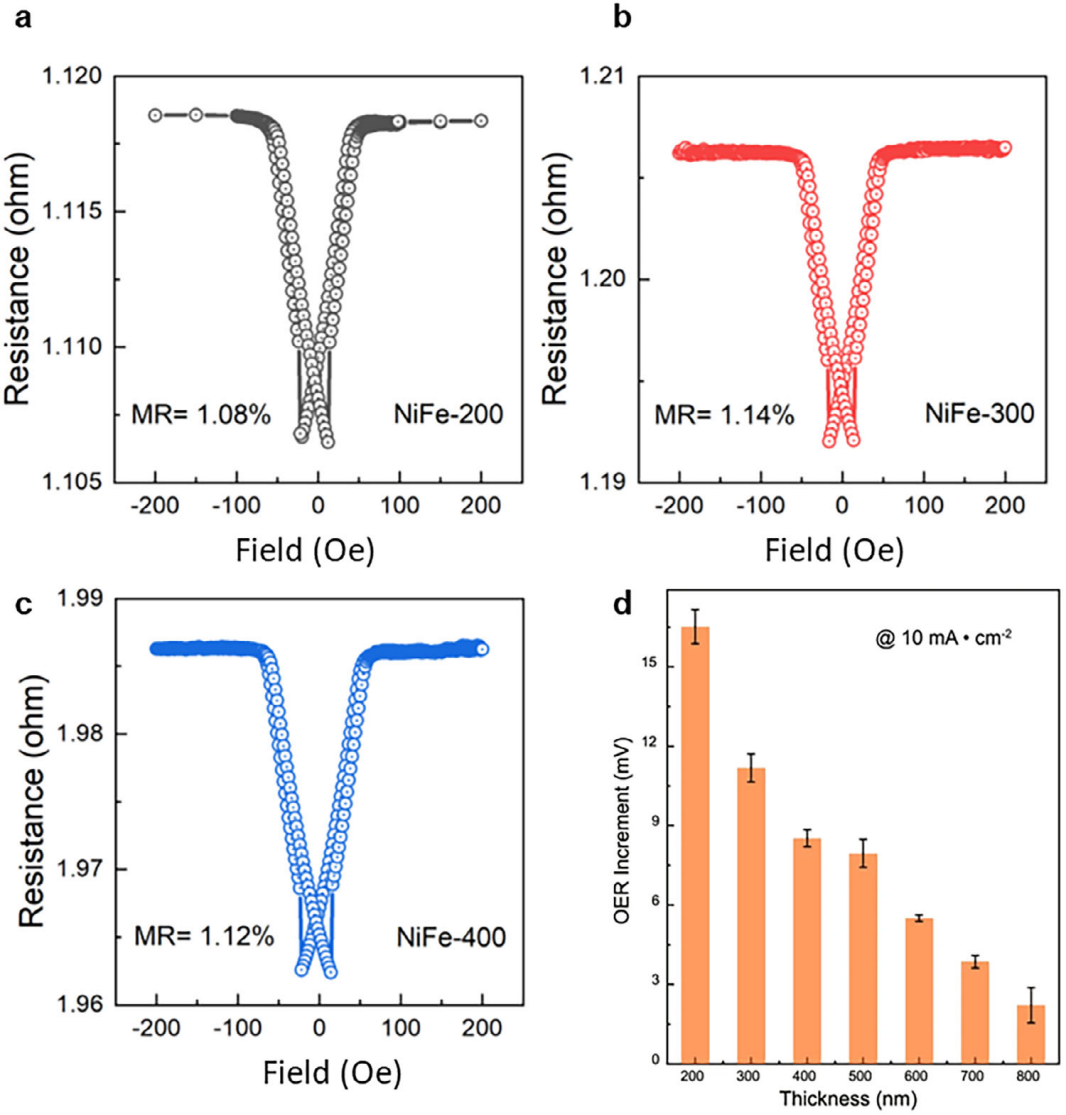
In‐plane magnetoresistance measurements for NiFe thin films with varying thicknesses a) 200 nm b) 300 nm and c) 400 nm. d) Decrease of overpotential at 10 mA cm^−2^ (in 1 m KOH under 200 mT fields) for increasing thickness of NiFe thin films from 200 to 800 nm, with the thinnest film showing the largest decrease. Reproduced with permission.^[^ ^50^ ^]^ Copyright 2023, Creative Commons Attribution 4.0 International License.

Interestingly, a material with pMR can still show enhancement under a magnetic field toward electrochemical OER. Ren et al. Showed decreased overpotential at 10 mA cm^−2^ for a range of NiFe thin film catalysts with varying thickness (200–800 nm), even though small pMR was measured for them between −200 and 200 mT (**Figure** **8**). The improved performance is attributed to the disappearance of domain walls upon magnetization. The materials’ surface that was taken up by domain walls is turned into a single domain and can therefore participate in OER through spin‐facilitation.^[^ ^50^ ^]^ This highlights the importance of considering multiple mechanisms, as the observation of a trend in MR cannot solely justify a magnetic effect.

Unfortunately, MR is not considered or measured in most magneto‐electrocatalytic studies; however, as shown here, it can potentially play a dominating role for paramagnetic, ferromagnetic, and antiferromagnetic electrodes under an external magnetic field. Based on these findings, it is reasonable to expect that significant pMR of a material could even lead to negative effects in electrocatalytic OER. We should highlight the importance of also considering the electrode substrate, not just the surface catalyst, as these MR properties can also impact the overall magnetic effect.

The relationship between a catalyst material's magnetic properties and magnetic‐field‐enhanced OER performance remains complex, with multiple contributing mechanisms. The dominating mechanism can change between systems, based on catalyst properties and experimental set‐up. Previous work in the literature has demonstrated correlations between the catalyst's magnetization, magnetoresistance, and electrochemical activity under a magnetic field, but significant discrepancies exist in reported effects. Ferromagnetic materials have shown a clear ability to enhance OER under a magnetic field, but the ability of non‐magnetic materials remains controversial. The findings discussed in this section emphasize the need for comprehensive studies investigating intrinsic material properties and external field interactions.

### 3.2 Orientation and Lorentz Force

Lorentz force and hence, MHD, is ubiquitous in magneto‐electrocatalytic systems, but the direction, magnitude, and consequential catalytic effects are dependent on exact system parameters. Orientation of the external magnetic field is a particularly important consideration, as this determines how it interacts with both the inter‐ and intra‐electrode current density. Other reactor parameters will strongly influence the size of this magnetic effect, such as electrode geometry, electrode magnetization, inter‐electrode distance, and electrolyte concentration. This section evaluates Lorentz force effects in various set‐ups published in the literature.

**Figure 9:**
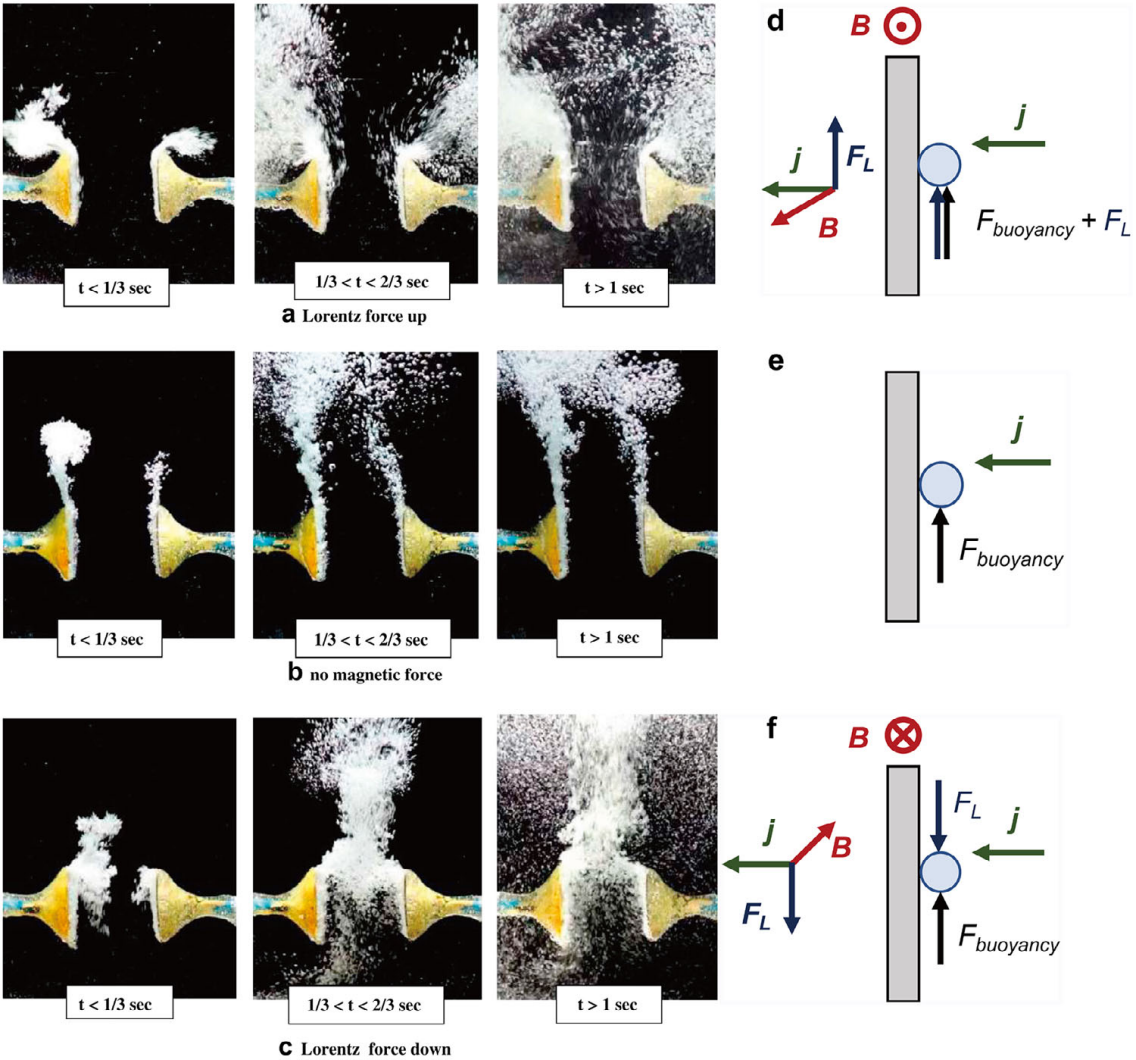
MHD effects on bubble movement using Ni plates as WE and CE when a) Lorentz force is upward, b) no Lorentz force is present and c) Lorentz force is downward. Schematic representations of d) upward F_L_ and F_buoyancy_ aiding bubble dispersion e) bubble movement is only controlled by F_buoyancy_ and f) F_L_ is opposing F_buoyancy_. Reproduced with permission.^[^ ^22^ ^]^ Copyright 2012, Elsevier.

Lin et al. built a small electrolytic cell for OER, where an external magnetic field can be applied perpendicular to the inter‐electrode current density using two commercial rubidium iron magnets. Fascinating images of MHD effects were recorded in a two‐electrode system using ferromagnetic nickel electrodes for water splitting when 4 V was applied. Photos taken at t < 1/3 s, 1/3 < t < 2/3 s and t > 1 s clearly show the effects of Lorentz force on bubble movement and mass transport at different orientations. **Figure** **9** shows the bubble movement for both H_2_ (left) and O_2_ (right) in three different cases. When upward Lorentz force is present (Figure 9a,d), bubble dispersion is aided through vigorous MHD (especially at t > 1 s), improving OER performance. The lack of magnetic field and therefore, no Lorentz force, results in bubble movement controlled by buoyancy force alone (Figure 9b,e). A downward Lorentz force results in downward movement of bubbles and restricts their removal from the interelectrode space, worsening performance (Figure 9c,f).^[^ ^22^ ^]^

In this unique study, the team also investigated changing the inter‐electrode distance. They found that when the inter‐electrode distance is reduced to 2 mm between the Ni electrodes, (4.5 T field, 20% KOH, 4 V, upward Lorentz force), the increase in current density under field was up to 246.5 mA cm^−2^, which decreased to ≈110 mA cm^−2^ at 5 mm inter‐electrode distance and ≈40 mA cm^−2^ at 10 mm. Electrode composition also played an important role in this study. The team used ferromagnetic Ni, paramagnetic Pt, and diamagnetic graphite as both WE and CE and found the order of current density enhancement to be Ni > Pt > graphite. This was the only paper we studied where there was no reference electrode used. A reference electrode allows for accurate control and stability of the potential at the WE surface, which should be considered when designing magneto‐electrocatalytic systems.

**Figure 10:**
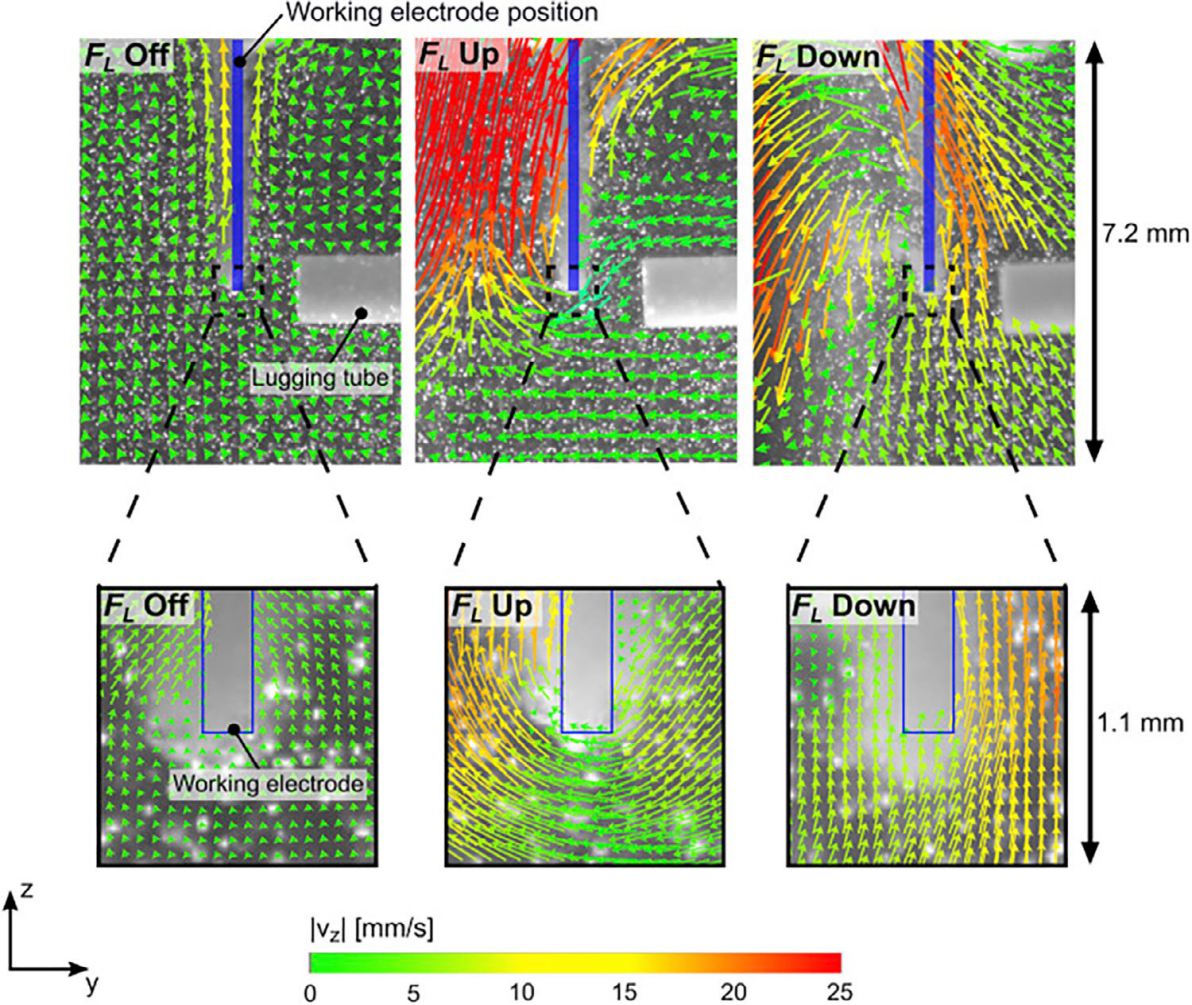
PIV results at 1 A cm^−2^ for Ni wire WE without magnetic field and in the presence of both upward and downward Lorentz forces. Reproduced with permission.^[^ ^28^ ^]^ Copyright 2024, Elsevier.

When the geometry of the electrode changes, the way MHD impacts mass transport can change. In a similar experiment by Fogaça et al., a cylindrical Ni wire WE with a thicker Ni wire CE, both upward and downward Lorentz force enhanced oxygen evolution. The main difference between this study and that by Lin et al., is in the shape of the electrodes: plate versus wire. Unique particle image velocimetry (PIV) experiments were conducted by the team, to visualize the effect of upward and downward Lorentz forces on bubble movement. **Figure** **10** shows the obtained PIV images, where the left side of each image is the inter‐electrode space. Without a magnetic field, the electrolyte moved upward due to the upward movement of bubbles – from buoyancy force. When an upward Lorentz force was present, the speed of upward electrolyte movement significantly increased in the inter‐electrode space. Interestingly, when a downward Lorentz force was present, a clear downward movement was observed in the inter‐electrode space, which changes to an upward movement on the electrode surface. Both directions of Lorentz force caused a reduction of the overpotential, with downward Lorentz force resulting in the best performance due to more chaotic convection and enhanced bubble detachment.^[^ ^28^ ^]^

**Figure 11:**
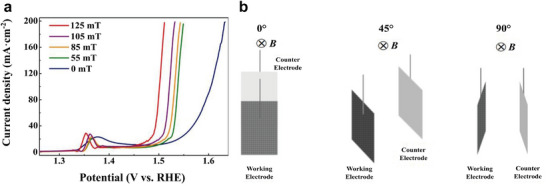
a) LSV curves recorded for Co_3_O_4_ on Ni foam with and without 125 mT magnetic field at different orientations. b) Schematic illustration of the 3 orientations tested with the relative position of the WE and CE to the magnetic field. Figures (a) and (b) are reproduced with permission.^[^ ^53^ ^]^ Copyright 2019, Elsevier.

Investigating different orientations, Li et al. studied the change in LSV curves in three orientations: 0° (magnetic field parallel to inter‐electrode current density), 45° and 90° (magnetic field perpendicular to inter‐electrode current density). Performance of the Co_3_O_4_/ Ni foam catalyst increased in all three orientations compared to the baseline experiment run without a magnetic field present. The largest enhancement was observed for the 90° orientation (upward Lorentz force is present), where the overpotential was reduced by ≈140 mV. An enhancement of performance is observed as this Lorentz force increases due to increased MHD (**Figure** **11**a,b).^[^ ^53^ ^]^

MHD effects induced by Lorentz force have been shown to significantly affect mass transport. It influences the movement of both the reactive species toward the electrode and the products away from it. Since MHD effects are directly induced by Lorentz force, they are strongly dependent on orientation. Other influential factors are electrode shape, material, and reactor setup. Plate‐shaped electrodes have been shown to be more susceptible to negative effects from downward Lorentz forces, whereas a wire‐shaped electrode is more susceptible to positive effects due to chaotic convection from any direction of Lorentz force. This may stem from a more directional inter‐electrode electric field and therefore unidirectional Lorentz force between plate‐shaped electrodes; but, also will depend on the natural bubble movement about each electrode shape. Reactor dimensions including electrode positions and distances are also important, as these factors impact the strength of the inter‐electrode electric field from that the Lorentz force is derived.

### 3.3 Considerations of Electrolyte pH

OER is a pH‐dependent reaction, as discussed above in Section 1. Therefore, the dependency of the magneto‐electrocatalytic effect on pH an important and interesting point of investigation.

**Figure 12:**
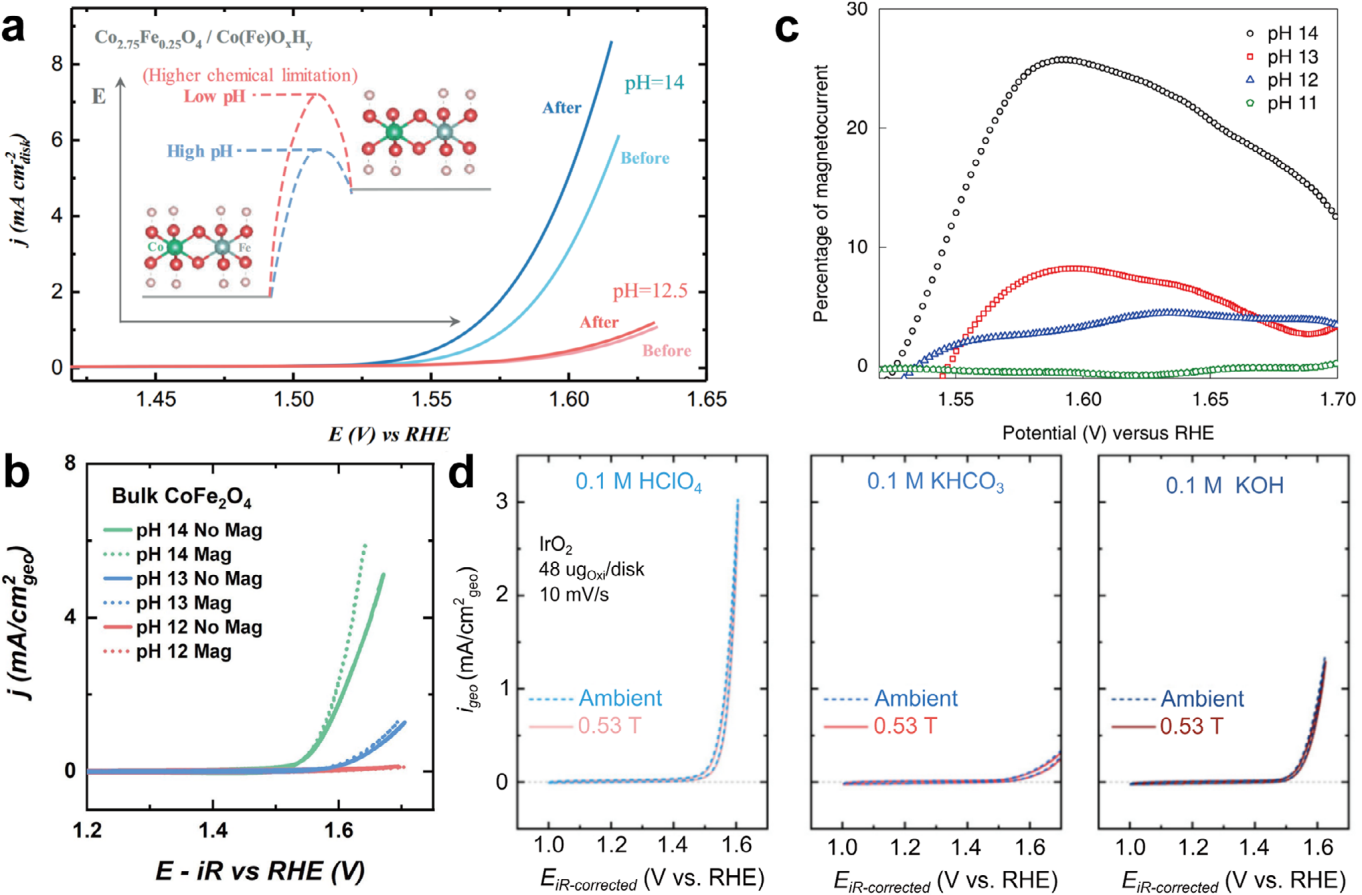
a) LSV curves of reconstructed Co_2.75_Fe_0.25_O_4_ (s) in pH 12.5 and 14 KOH with and without 500 mT magnetic field. Reproduced with permission.^[^ ^34^ ^]^ Copyright 2021, Creative Commons Attribution 4.0 International License. b) LSV data for CoFe_2_O_4_ with and without magnetization in various pH electrolytes. Reproduced with permission.^[^ ^54^ ^]^ Copyright 2023, Wiley. c) Percentage magnetocurrent as a function of applied potential at various pHs of KOH electrolyte under ≤450 mT field. Reproduced with permission.^[^ ^38^ ^]^ Copyright 2019, Springer Nature. d) CV scans for commercial IrO_2_ in electrolytes with different pH values with and without 530 mT field. Reproduced with permission.^[^ ^55^ ^]^ Copyright 2022, Elsevier.

The size of the magnetic effect was found to be heavily pH dependent: high pH electrolytes show much greater enhancement, that becomes almost negligible as the pH drops. This is attributed to a high concentration of OH^‐^ facilitating deprotonation and generation of the oxyl intermediates.^[^ ^34^, ^38^, ^54^ ^]^ In the case of various ferromagnetic catalysts (**Figure** **12**a,b), the size of the magnetic effect was found to be heavily pH dependent: high pH electrolytes show much greater enhancement, which becomes almost negligible as the pH drops. This is attributed to the high concentration of OH^‐^ facilitating deprotonation and generation of the oxyl intermediates.^[^ ^34^, ^38^, ^54^ ^]^

Wu et al. reported a notable enhancement for reconstructed Co_2.75_Fe_0.25_O_4_ catalyst under 500 mT field in pH 14 KOH electrolyte, which becomes very limited as the pH is decreased to 12.5 (Figure 12a).^[^ ^34^ ^]^ This result is in agreement with the work done by Ge et al. On CoFe_2_O_4_ catalyst, where the magnetic enhancement is significant at pH 14 even at very low current densities, it becomes negligible as pH drops to 12 (Figure 12b).

Interestingly, Garcés‐Pineda et al. found that the percentage change in current density at a given pH under a magnetic field does not scale with the applied potential due to chaotic bubbling at the electrode surface, and a decrease is observed at higher potentials (Figure 12c). In a recent work by Wei and Xu, no change was observed in the performance of IrO_2_ with and without 530 mT field in electrolytes of pH 0.9 (0.1 m HClO_4_), 7.8 (0.1 m KHCO_3_) or 12.8 (0.1 m KOH) (Figure 12d).^[^ ^55^ ^]^ All LSV and CV curves presented in Figure 12 have been *iR* corrected to adjust for the change in solution resistance of electrolytes with different pH.

The pH‐dependency of the magneto‐electrocatalytic effect on OER highlights a crucial factor in optimizing OER performance for practical applications. Systems with high pH electrolytes exhibit significant under a magnetic field due to the role of OH^−^ facilitating the generation of key intermediates. The findings described in this section highlight the importance of electrolyte selection before scale‐up of magneto‐electrocatalytic systems. Investigations are needed on the interplay between electrolyte pH, a catalyst‘s properties, and resulting magnetic field effects.

### 3.4 Difficulties of Non‐Standard Reaction Setups

Here, we discuss experimental design choices that are not commonly observed in magento‐electrocatalytic OER studies, including the use of non‐vertical electrodes, non‐uniform fields, and placing only the WE into the magnetic field.

**Figure 13:**
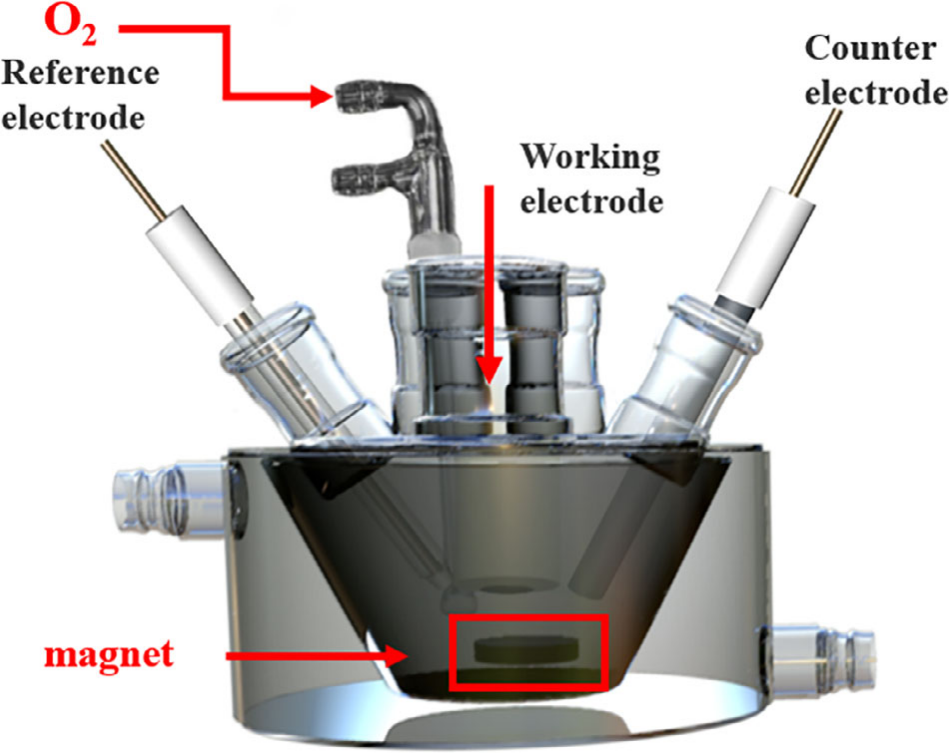
Schematic illustration of a reactor set‐up where the glassy carbon WE is pointing vertically downward and the magnet is placed inside the electrolyte. Reproduced with permission.^[^ ^56^ ^]^ Copyright 2022, Springer Nature.

Most common electrodes used in magneto‐electrocatalytic OER are plate‐shaped sitting vertically (as summarized in Table 1) and therefore the direction of upward bubble movement governed by buoyancy force is obvious. Glassy carbon electrodes are sometimes used as substrates in magneto‐electrocatalytic OER studies (Table 1).^[^ ^34^, ^46^, ^47^, ^48^ ^]^ In work by Qin et al., a glassy carbon, rotating disk electrode coated with NiFe LDH is placed to face the reactor bottom (shaped (as summarized in Table 1) and therefore the direction of upward bubble movement governed by buoyancy force is obvious. Glassy carbon electrodes are sometimes used as substrates in magneto‐electrocatalytic OER studies (Table 1).^[^ ^34^, ^46^, ^47^, ^48^ ^]^ In the work by Qin et al., a glassy carbon, rotating disk electrode coated with NiFe LDH is placed to face the reactor bottom (**Figure** **13**). In this vertical position, the efficiency of cooperative buoyancy and Lorentz forces will change when as generated bubbles will need to move sideways to be removed from the electrode surface. To facilitate the mass transport of bubbles, a rotating disk electrode is often used in electrochemistry. The use of a rotating disk electrode will constantly change the location of an active site within the magnetic field, potentially making it harder to understand the source of the enhancement and raising questions on whether a small field can really enhance mass transport when the electrode is already rotating at significant speeds.^[^ ^47^, ^56^ ^]^

Here, we would like to highlight the importance of accurate reporting of reproducible experimental data. As reported by Hunt et al., even a small change in the relative position of the working electrode to the magnet will result in a significant change in the magnetic flux density on the surface.^[^ ^37^ ^]^ Some studies in the literature would benefit from a more robust investigations into reactor design. For example, placing magnets inside the electrolytic cell (Table 1) without ensuring the stability of their position between experiments, maintaining a consistent distance between the WE surface and the magnet, and preventing magnet corrosion or metal dissolution into the electrolyte can lead to unreliable or non‐reproducible data (Figure 13).

In this digital age, attaching supplementary videos can be a helpful way of demonstrating magnetic effects on water splitting (e.g., through showing an instant increase in the rate of O_2_ and H_2_ bubble formation). They can also reveal inconsistencies in the reaction setup with and without the magnetic field. Videos in the work by Garcés‐Pineda et al. reveal movement of the electrode on magnetic field application. The changes in electrode position will affect the inter‐electrode field and therefore, the catalytic performance. Consequentially, doubt is thrown upon the reliability of the magnetic effects reported in this paper.^[^ ^38^ ^]^

**Figure 14:**
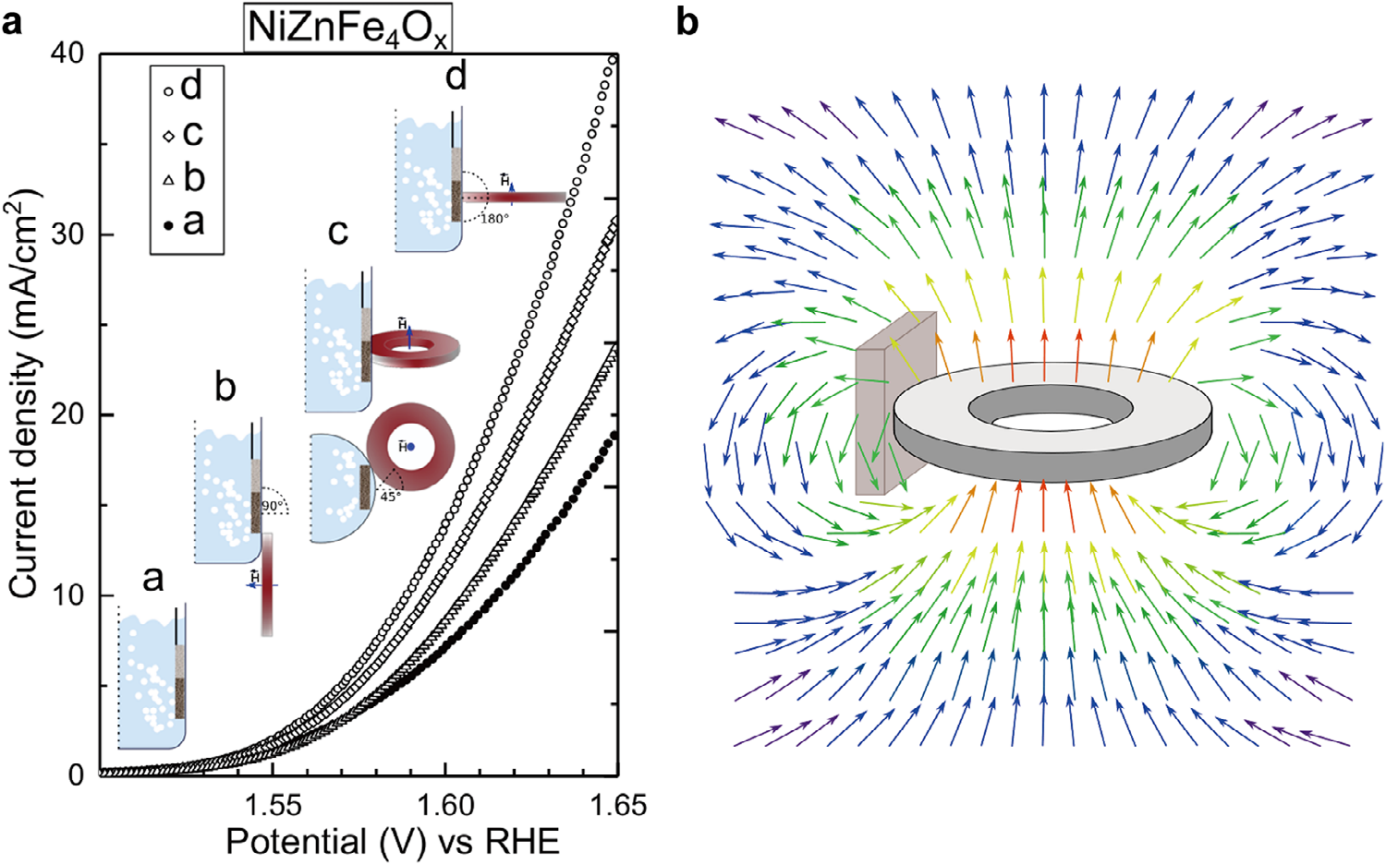
a) LSV curves of NiZnFe_4_O_x_ on Ni foil under no magnetic field and under a magnetic field with various orientations of the permanent Nd ring magnet. b) Illustration of the magnetic field force lines around the magnet. Reproduced with permission.^[^ ^38^ ^]^ Copyright 2019, Springer Nature.

By placing the reactor between two magnets, the magnetic field lines become straight and align perpendicularly to the magnet surface; making the field direction easy to predict. Garcés‐Pineda et al. used a single Nd magnet ring and studied the effect of changing its orientation including a useful illustration of field lines. Since the electrode here is placed in a non‐uniform field, Lorentz force directions are more challenging to determine (**Figure** **14** ), which makes the orientation‐study less reliable.^[^ ^38^ ^]^

Another unique experimental set‐up design strategy is placing only the WE in the magnetic field, rather than the entire reactor, through the use of 2 or 3‐compartment electrochemical cells (summarized in Table 1). This allows for investigations on the magnetic field effects on the WE only, without affecting the RE or CE.^[^ ^39^, ^51^, ^53^, ^55^, ^56^ ^]^ As discussed above, there is still no agreement on how the physical properties of an electrode material affect their response to the magnetic field. The most commonly used CEs are platinum (paramagnetic) or graphite (diamagnetic) based materials with varying shapes (Table 1).^[^ ^61^, ^62^ ^]^ We believe future research should investigate the different effects observed when all or only one electrode is placed inside the magnetic field. Publishing detailed experimental methods, including reactor parameters and inter‐electrode distances, is crucial to allow researchers to build on existing literature by changing only one parameter at a time.

Since both current flow and Lorentz force are directional, the relative orientation and uniformity of the magnetic field are crucial details for designing magneto‐electrochemical set‐ups. While many reports have demonstrated improved performances is many different field orientations, the reporting of accurate and reproducible experimental data remains crucial. Even small variations in the electrode positioning and reactor design can significantly impact the observed results, emphasizing the need for standardized methods. Further research should focus on refining experimental set‐ups and their effect on the observed magnetic enhancement.

### 3.5 Quantification of Magnetic Effects

We believe that the greatest challenge faced by researchers in the field is the lack of consistency across different studies. It is hard to find two papers whose experimental set‐ups – catalyst, WE preparation, reactor design, magnet type, magnetic field strengths – are similar to a level where comparison is easy. So far, we have focused on investigating how system design choices affect the magnetic effect observed in catalysts. In this section, we discuss some methods commonly used to quantify the magnetic effect.

**Figure 15:**
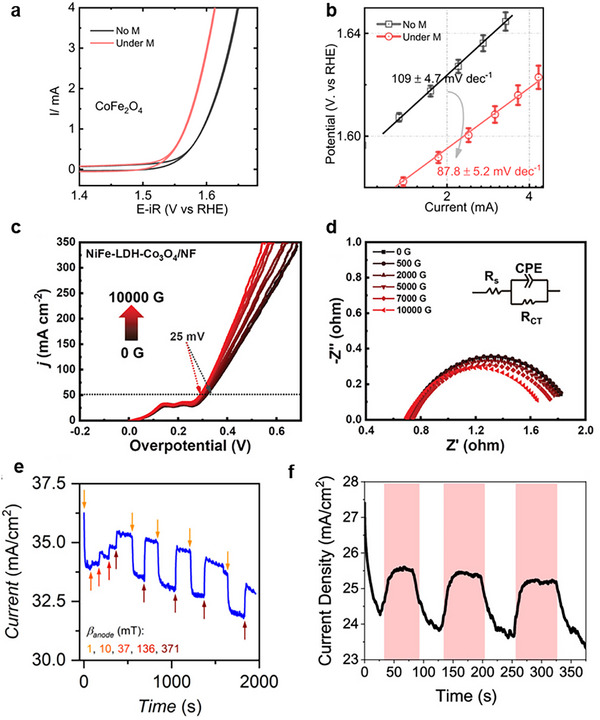
CV scans for CoFe_2_O_4_ with *iR* correction in 1 m KOH with and without 1 T applied field. Reproduced with permission.^[^ ^46^ ^]^ Copyright 2021, Creative Commons Attribution 4.0 International License. LSV curves without *iR* correction c) and EIS data d) for NiFe‐LDH‐Co_3_O_4_ on Ni foam catalyst in 1 m KOH in increasing magnetic fields between 0 and 1 T. Reproduced with permission.^[^ ^49^ ^]^ Copyright 2022, Wiley. e) CA graph obtained for anodized CoO_x_ on FTO in 0.1 m KOH at 1.5 V versus NHE under different magnetic fields. Reproduced with permission.^[^ ^37^ ^]^ Copyright 2022, American Chemical Society. f) CA graph for a similar treated CoO_x_ thin fil catalyst on FTO in 0.1 m KOH at 0.9 V versus NHE with 400 mT magnetic field pulses (highlighted in pink). Reproduced with permission.^[^ ^36^ ^]^ Copyright 2020, American Chemical Society.

We have found better consistency in the types of electrochemical techniques used for quantification of the magnetic effect. Linear sweep voltammetry and cyclic voltammetry are standard techniques used to evaluate the performances of materials over a range of applied potentials, and calculate Tafel slopes and overpotentials (**Figure** **15**a,b). In certain cases, the magnetic field effect becomes significantly more apparent at higher applied potentials. For example, Figure 15c shows negligible effects ≈10 mA cm^−2^, but the magnetic enhancement becomes greater above 50 mA cm^−2^.

Electrochemical impedance spectroscopy with and without a magnetic field is commonly used to study the charge transfer and solution resistances with and without the presence of a magnetic field (Figure 15d) and the data obtained can be used for *iR* correction of LSV and CV curves. Not all data presented in the literatures uses *iR* correction or specify if they did or did not, and for the ones that do, the level of correction ranges from 20% to 100%.^[^ ^50^, ^56^ ^]^ This discrepancy calls attention to the importance of clear and comprehensive reporting of experimental procedures but also highlights the issues presented when one tries to compare different studies.

Chrono techniques (chronoamperometry (CA) or chronopotentiometry (CP)) can allow for instant monitoring of a system's response to an applied field and for an easy and convincing demonstration of the magnetic effect (Figure 15e,f). We believe these measurements should be a standard element of all magneto‐electrocatalytic papers.

A major challenge in this field of research is the lack of consistency in experimental set‐ups, making direct comparisons between studies difficult. While system design choices tend to be unique for each study, greater uniformity exists in the electrochemical techniques used for quantification. LSV and CV remain standard methods for evaluating performance under normal conditions and with the applied external magnetic field. EIS provides valuable insights into the resistance associated with a particular set‐up and results are commonly used for *iR* correction, though inconsistencies in correction levels complicate the comparison of studies. Chrono techniques offer a real‐time approach to demonstrating magnetic effects. These findings emphasize the need for standardized methodologies and transparent reporting to advance the field and enable meaningful comparisons between studies.

## 4 Challenges

This field has many challenges that come from: the availability of magnetic field sources, incomprehensive mechanistic analysis, and incompatibility of many standard analytical techniques with strong magnetic fields.

The range of magnetic field sources available to researchers can make the repeatability and cohesion of experiments difficult. Particular issues arise when only one magnet is used, which makes the direction of field lines difficult to predict; limiting the reliability of Lorentz force assessments. Ideally, the reactor should be placed inside a large electromagnet that can produce parallel and directional magnetic field lines. The magnetic field strength can then easily be controlled by adjusting the power applied to the electromagnet and should be measured with a Gauss meter.

Simultaneous action of different mechanisms naturally makes magneto‐electrocatalytic systems difficult to interpret. Untangling these mechanisms will require significant experimental innovation. Major challenges exist in conducting standard in situ chemical analyses within strong magnetic fields. These analytical techniques are essential for the full understanding of Lorentz force on intra‐electrode charge movement, and to confirm triplet O_2_ promotion at active sites. Additionally, bubble imaging cameras will need to be integrated into various reactor setups to understand the MHD intricacies.

Due to these difficulties, some papers choose to assess only one possible mechanism, despite the likelihood of interacting mechanisms. This can lead to a misconception or over‐reporting of one mechanism that does not accurately reflect the truth of magneto‐electrocatalytic systems.

It's important to realize that magnetic fields can cause negative effects on performance. There are very few reports detailing this, likely due to the general academic aversion to publishing negative results. However, if we do not understand these negative mechanisms, we cannot build systems that purposefully limit their impact.

Without addressing these problems, we cannot move closer to understanding how to develop systems toward different effects, in order to realize the maximum magnetic enhancement on electrocatalytic water‐splitting.

## 5 Future Perspectives

The application of an external magnetic field in electrochemical water‐splitting is a promising way to improve the efficiency of the overall reaction. Through optimization of systems toward maximum magnetic enhancement, the technique will have real commercial promise for efficient green‐H_2_ production.

After catalyst design is optimized, engineers will need to consider how to integrate magnets into industrial electro‐catalytic reactors, particularly considering the compatibility of electrical components. It'll also be important to consider if the catalyst optimized toward magneto‐electric effects will meet a set of criteria regarding its affinity toward OER, sustainability, and cost efficiency. Life cycle assessments and techno‐economic analyses need to be conducted to determine the feasibility of the process. These can be built on existing assessments of two main components of the process, magnets, and electrolyzers, which are already present in the industry. The use of electromagnets or permanent magnets as the field source will also need to be evaluated.

The use of magnetic fields may also have applications for standard water electrolyzers, currently used in industry. Lorentz force could be exploited to move H_2_ and O_2_ to separate parts of the reactor, simplifying gas separation and removing the need for expensive membranes.

## 6 Conclusion

This perspective explores the relationship between fundamental chemistries in magneto‐electrochemical OER and experimental design for the optimization of such systems for maximum positive effects. Key findings indicate that it is essential to investigate all possible mechanisms for a given system with both in situ electrochemical characterization techniques with and without magnetic fields and extensive ex situ analysis of the materials with techniques such as magnetization and magnetoresistance measurements. In situ electrochemical tests need to consider the orientations and magnitudes of the external magnetic and inter‐electrode electric fields. Varying the orientation and shape of the electrodes can pinpoint Lorentz force effects and aid the understanding of the dominating mechanism for a given system. It has been noted that, while reactor dimensions and electrode shape primarily influence Lorentz force‐driven MHD effects, spin‐polarization‐derived MR and triplet oxygen promotion have a greater dependence on the catalyst composition, availability of compatible active sites, and their magnetic properties.

Importantly, this perspective highlights the need for researchers to account for all potential magnetic effects, rather than selectively focusing on individual phenomena. MHD effects, for instance, are likely underappreciated in many reported systems due to insufficient investigations into changing reactor orientations relative to the magnetic field direction and therefore impacting Lorentz force directions. Moving forward, future studies should consider all these diverse effects to understand their synergistic or antagonistic interactions fully. Such an approach will be crucial for designing more efficient systems for magneto‐electrochemical water splitting, pushing the boundaries of current technology to achieve practical, scalable solutions for H_2_ production.

## Conflict of Interest

The authors declare no conflict of interest.

## Author Contributions

D.S. and A.R. contributed equally to this work. D.S. and A.R. wrote the manuscript in discussion with Y.L. SCET supervised the project.
